# Supplementary material for: ProteinShader: illustrative rendering of macromolecules
Source: BMC Struct Biol. 2009 Mar 30;9:19. doi: 10.1186/1472-6807-9-19 (PMC2672931; doi:10.1186/1472-6807-9-19)
Supplement: Additional file 1 — ProteinShader program without source code. This compressed file contains the complete ProteinShader program including associated libraries, but no source code. A README.txt file gives an overview of the ProteinShader distribution, and the index.html file in the help subdirectory has directions on getting started with the program as well as a set of tutorials. [file 1472-6807-9-19-S1.zip › ProteinShader-beta-0_9_4-binary/help/api/org/proteinshader/gui/components/controlpanel/class-use/ColorPanel.html]

Uses of Class org.proteinshader.gui.components.controlpanel.ColorPanel (ProteinShader API)


|  |  |  |
| --- | --- | --- |
| **Overview** \| **Package** \| **Class** \| **Use** \| **Tree** \| **Deprecated** \| **Index** \| **Help** | |  |
| PREV   NEXT | **FRAMES**    **NO FRAMES**     **All Classes** |  |


---


## **Uses of Class org.proteinshader.gui.components.controlpanel.ColorPanel**

| Packages that use ColorPanel | |
| --- | --- |
| **org.proteinshader.gui.components.controlpanel** | Holds the JPanels used in the right-side retractable control panel of the GUI. |
| **org.proteinshader.gui.listeners.controlpanel** | Holds the listeners for components used in the JPanels of the right-side retractable control panel of the GUI. |

Uses of ColorPanel in org.proteinshader.gui.components.controlpanel

| Subclasses of ColorPanel in org.proteinshader.gui.components.controlpanel | |
| --- | --- |
| `class` | `AtomColorPanel`             This control panel allows the user to modify the color of Atoms. |
| `class` | `CartoonColorPanel`             This control panel allows the user to modify the color of Segments. |

Uses of ColorPanel in org.proteinshader.gui.listeners.controlpanel

Constructors in org.proteinshader.gui.listeners.controlpanel with parameters of type ColorPanel

`ColorPanelListenerFactory(Mediator mediator,
ColorPanel colorPanel)`
  
          Constructs a ColorPanelListenerFactory.

---


|  |  |  |
| --- | --- | --- |
| **Overview** \| **Package** \| **Class** \| **Use** \| **Tree** \| **Deprecated** \| **Index** \| **Help** | |  |
| PREV   NEXT | **FRAMES**    **NO FRAMES**     **All Classes** |  |


---

# *Copyright © 2007-2008*
